# Mesenchymal Stem Cell-Derived Factors Restore Function to Human Frataxin-Deficient Cells

**DOI:** 10.1007/s12311-017-0860-y

**Published:** 2017-04-29

**Authors:** Kevin Kemp, Rimi Dey, Amelia Cook, Neil Scolding, Alastair Wilkins

**Affiliations:** Multiple Sclerosis and Stem Cell Group, School of Clinical Sciences, Clinical Neurosciences office, University of Bristol, 1st floor, Learning and Research building, Southmead Hospital, Bristol, BS10 5NB UK

**Keywords:** Friedreich’s ataxia, Mesenchymal stem cells, Frataxin, Oxidative stress

## Abstract

Friedreich’s ataxia is an inherited neurological disorder characterised by mitochondrial dysfunction and increased susceptibility to oxidative stress. At present, no therapy has been shown to reduce disease progression. Strategies being trialled to treat Friedreich’s ataxia include drugs that improve mitochondrial function and reduce oxidative injury. In addition, stem cells have been investigated as a potential therapeutic approach. We have used siRNA-induced knockdown of frataxin in SH-SY5Y cells as an in vitro cellular model for Friedreich’s ataxia. Knockdown of frataxin protein expression to levels detected in patients with the disorder was achieved, leading to decreased cellular viability, increased susceptibility to hydrogen peroxide-induced oxidative stress, dysregulation of key anti-oxidant molecules and deficiencies in both cell proliferation and differentiation. Bone marrow stem cells are being investigated extensively as potential treatments for a wide range of neurological disorders, including Friedreich’s ataxia. The potential neuroprotective effects of bone marrow-derived mesenchymal stem cells were therefore studied using our frataxin-deficient cell model. Soluble factors secreted by mesenchymal stem cells protected against cellular changes induced by frataxin deficiency, leading to restoration in frataxin levels and anti-oxidant defences, improved survival against oxidative stress and stimulated both cell proliferation and differentiation down the Schwann cell lineage. The demonstration that mesenchymal stem cell-derived factors can restore cellular homeostasis and function to frataxin-deficient cells further suggests that they may have potential therapeutic benefits for patients with Friedreich’s ataxia.

## Introduction

Friedreich’s ataxia (FA) is a genetic disorder caused by a GAA repeat expansion within the first intron of the frataxin gene (*FXN*) and is characterised by a number of clinical manifestations, including cerebellar ataxia and cardiomyopathy [1]. This expansion leads to reduced expression of frataxin, a mitochondrial protein the functions of which include iron chaperoning in iron-sulphur cluster and heme biosynthesis, maintenance of anti-oxidant defences and iron detoxification [2, 3]. The amount of frataxin gene and protein expression in clinically symptomatic patients varies from approximately 5–35% of normal levels. Furthermore, a correlation between reduced frataxin levels (longer GAA expansions) and earlier-onset neurological disease implies a role for frataxin in maintenance and protection of neurons [4]. The precise subsequent mechanisms of cell death and neurodegeneration remain the subject of active research, with an increasing consensus suggesting that oxidative damage plays a key role [5].

Cellular and in vivo models of frataxin deficiency show increased susceptibility to damage from reactive oxygen and nitrogen species, particularly hydrogen peroxide [6]. Cells derived from patients with FA are known to have deficiencies in defences against oxidative stress, and this is thought to be one of the major causes of tissue injury in the disease [7]. Reduction in cellular frataxin has been associated with decreases in superoxide dismutase (SOD) activity and hydrogen peroxide-detoxifying enzymes [8, 9]. This failure of endogenous anti-oxidant defences causes elevation in lipid peroxidation, mitochondrial dysfunction and DNA injury, with subsequent cytotoxicity and tissue damage [10]. Thus, one potential approach to reduce tissue injury in FA is to define therapies that boost endogenous frataxin and/or anti-oxidant responses.

Mesenchymal stem cells (MSCs) are being investigated extensively as potential treatments for a wide range of diseases, not least neurological disorders. The mode of action of stem cell-mediated neuroprotection is likely to be multifactorial, but boosting endogenous repair mechanisms in disease states, including FA [7, 9, 11, 12], is a key area for research. Indeed, increasing evidence implies the major mechanistic neuroprotective role of MSCs is their capacity to secrete a diverse range of potentially neuroprotective factors or through anti-oxidant actions [13–15]. If stem cell therapies are to become a viable therapeutic approach for central nervous system (CNS) degeneration, a precise understanding of their effects on neurons is required.

With this aim, we have modelled frataxin-knockdown in SH-SY5Y cells to both further understand oxidative stress mechanisms within frataxin-deficient cells and investigate the potential anti-oxidant properties of MSC-secreted factors. SH-SY5Y cell lines are composed of multipotent precursor cells that give rise to distinct neural crest cell lineages and have been widely used for studies of neurodegenerative disorders [16]. The SH-SY5Y cell line may have particular relevance to FA; cells affected in FA including neurons of the dorsal root ganglion (DRG), satellite cells, Schwann cells and axons of sensory nerves and dorsal spinal roots all derive from the neural crest. Thus, pathophysiology of FA may be attributed to defects in shared precursor cells [17]. Here, we show that frataxin-deficient SH-SY5Y cells have reduced anti-oxidant defences, increased sensitivity to hydrogen peroxide-mediated toxicity and abnormalities in both cell proliferation and differentiation. Furthermore, exposing frataxin-deficient SH-SY5Y cells to MSC-secreted factors restores frataxin expression, anti-oxidant homeostasis and cellular function.

## Materials and Methods

### Bone Marrow Mononuclear Cell Isolation from Healthy Control Patients

Bone marrow samples were obtained by an orthopaedic surgeon at Southmead Hospital, Bristol, UK, with informed written consent and hospital ethic committee approval by the North Bristol NHS trust research ethics committee. Bone marrow was taken at the time of total hip replacement surgery from the femoral shaft and placed into a sterile 50-ml tube containing 1000 IU heparin. Patients with a history of malignancy, immune disorders or rheumatoid arthritis were excluded from the study. Femoral shaft bone marrow donors were healthy apart from osteoarthritis and were not receiving drugs known to be associated with myelosuppression or bone marrow failure.

Femoral shaft marrow samples were broken up with a scalpel and washed with Dulbecco’s modified Eagle’s medium (DMEM) (Sigma-Aldrich, Gillingham, UK) until remaining material, the bone looked white at the bottom of the 50-ml tube. All washings were pipetted into a new 50-ml tube and kept for centrifugation. The suspension was centrifuged and re-suspended in DMEM and overlaid onto an equal volume of Lymphoprep (Axis-Shield, Dundee, UK; density 1.077 ± 0.001 g/ml) and centrifuged at 600×*g* for 35 min at room temperature to separate the mononuclear cells from neutrophils and red cells. The mononuclear cell layer was harvested and washed twice in DMEM.

### Mesenchymal Stem Cell Culture

Mononuclear cells were isolated, centrifuged and re-suspended in MSC medium consisting of DMEM with 10% foetal calf serum (FCS) selected for the growth of MSCs (Stem Cell Technologies, London, UK), 2% L-glutamine and 1% penicillin and streptomycin (Sigma-Aldrich, Gillingham, UK). For the primary culture, vented flasks (25 cm^2^) containing 10 ml of MSC medium were seeded with 1 × 10^7^ cells and then incubated in a humidified atmosphere at 37 °C containing 5% CO_2._ These cells were fed every week with MSC medium by half medium exchange to remove non-adherent haematopoietic cells until the adherent fibroblast-like MSCs reached approximately 70% confluence. On reaching confluence, the adherent cells were re-suspended using 0.25% trypsin-EDTA (Sigma-Aldrich, Gillingham, UK) and re-seeded at 2.25 × 10^5^ cells per (75 cm^2^) flask into first passage. Cultures were then incubated, fed every week with MSC medium by half medium exchange and again trypsinised, a cell count taken and re-seeded at 2.25 × 10^5^ cells per flask (75 cm^2^). Cell cultures were incubated at 37 °C containing 5% CO_2_. At 2–3 passage MSCs were phenotypically characterised using cell surface marker expression and differentiation protocols as described in previous studies from our laboratory [15, 18, 19].

### Mesenchymal Stem Cell Conditioned Medium

Confluent MSCs, at passages 3–5, were washed in DMEM and cultured for 24 h in minimal base medium consisting of DMEM (Stem Cell Technologies, London, UK) supplemented with 1% insulin-free Sato (containing 100 μg/ml bovine serum albumin, 100 μg/ml transferrin, 0.06 μg/ml progesterone, 16 μg/ml putrescine, 0.04 μg/ml selenite, 0.04 μg/ml thyroxine, 0.04 μg/ml tri-iodothryonine), 1% holo-transferrin, 1% penicillin/streptomycin and 0.5% L-glutamine (Sigma-Aldrich, Gillingham, UK). The medium was then removed prior to being used within culture experiments. MSC conditioned medium in this study was prepared from >10 independent MSC samples.

### SH-SY5Y Cell Culture

Human neuroblastoma SH-SY5Y cells (Obtained from the European Collection of Authenticated Cell Cultures (ECACC)) were grown in SH-SY5Y growth medium DMEM (glucose 4 g/l) (Sigma-Aldrich, UK) with 10% FCS (Sigma-Aldrich, Gillingham, UK), 2% L-glutamine (Sigma-Aldrich, Gillingham, UK) and 1% penicillin and streptomycin (Sigma-Aldrich, Gillingham, UK). These cells were fed every 48 h and passaged once the cells reached a confluency of approximately 80%. Adherent cells were incubated with 0.25% trypsin-EDTA (Sigma-Aldrich, Gillingham, UK) at 37 °C with 5% CO_2_ for 5 min in order to dissociate adherent cells from the culture flasks. Proteolysis by trypsin was stopped with the addition of DMEM supplemented with 10% FCS. The dissociated cells were centrifuged with the SH-SY5Y culture medium at 600×*g* for 10 min before being counted and seeded into fresh culture flasks/wells. Cell cultures were incubated at 37 °C containing 5% CO_2_. Cells were not cultured past passage 25.

### Transfection of SH-SY5Y Cells

Transfection of SH-SY5Y was carried out using Amaxa Nucleofector cell line kit V (Lonza, Basel, Switzerland). 4 × 10^6^ cells were transfected with *FXN* small interfering RNA (siRNA) (diluted in water) (Applied Biosystems, UK) according to the manufacturer’s instructions. The nucleofector programme G-04 was chosen for high transfection efficiency. Cells were subsequently re-suspended in DMEM/10% FCS/2% L-glutamine and seeded into 6- or 96-well plates at a density of 5 × 10^5^ per well and 1 × 10^4^ per well, respectively, for further experimentation. For a mock-treated control, distilled water (H_2_O carrier transfected control) or scrambled siRNA (Applied Biosystems, UK) was used in place of the *FXN* siRNA.

### SH-SY5Y Expansion and Differentiation

For expansion, transfected or non-transfected cells were cultured for 24 h, trypsinised and re-seeded at 2 × 10^5^ in a 6-welled plate. Cells were cultured in either 2× SH-SY5Y growth medium/minimal medium (1:1) or 2× SH-SY5Y growth medium/MSC conditioned medium (1:1). Medium was replaced every 2 days and cells numerated after 8 days.

For differentiation, transfected or non-transfected cells were cultured for 24 h, trypsinised and re-seeded at 2 × 10^5^ in a 6-welled plate containing 10 μM retinoic acid (RA) (Sigma-Aldrich) in 2× SH-SY5Y growth medium/minimal medium (1:1) or 2× SH-SY5Y growth medium/MSC conditioned medium (1:1) for 8 days. Differentiation medium was replaced every 2 days. Control cells were treated identically but with an equal volume of vehicle ethanol (0.02%) in place of RA. Both SH-SY5Y differentiated Neuronal (N type) and Schwann (S type) cells were identified based on their distinct morphology.

### Western Blotting

SH-SY5Y cells were lysed using Beadlyte cell signalling universal lysis buffer (Upstate™, UK). To ensure there was equal loading of cell lysates, the concentration of total protein of each cell lysate sample was calculated using the Qubit® Fluorometer and Quant-iT™ protein assay kit (Invitrogen, UK) according to manufacturers’ instructions. Laemmli 2× sample buffer (Invitrogen, UK) was added to the lysates and then heated for 5 min at 95 °C. These samples were then run on Tris-HCL 4–20% ready gels (Bio-Rad, UK). Following transfer to a nitrocellulose membrane (Bio-Rad, UK) and blocking in Tris-buffered saline/5% bovine serum albumin, membranes were incubated in primary antibody overnight at 4 °C in Tris-buffered saline/5% bovine serum albumin. The antibodies used were the following: anti-β-actin (1:5000; Abcam, UK); rabbit anti-catalase (1:10,000; Abcam, UK); mouse anti-human frataxin (which recognises a band migrating at approximately 18 kDa corresponding to the mature frataxin protein isoform (aa 56–210) (1:1000; Millipore, UK) or mouse anti-human frataxin (1:1000 Abcam, UK); rabbit anti-glutathione peroxidase 1 (GPX) (1:5000; Abcam, UK); anti-nuclear factor (erythroid-derived 2)-like 2 (Nrf2) (Santa Cruz; 1:3000), anti-peroxisome proliferator-activated receptor gamma coactivator 1-alpha (PGC1a) (Santa Cruz; 1:3000); rabbit anti-superoxide dismutase 1 (SOD1) (1:4000); and mouse anti-superoxide dismutase 2 (SOD2) (1:4000; both Abcam, UK). For cell signalling studies, antibodies used were: phospho-Akt (Ser473); and total Akt (both 1:1000; Cell Signalling Technology). Secondary antibodies: anti-rabbit or anti-mouse horseradish peroxidase conjugated antibodies (in Tris-buffered saline/5% bovine serum albumin; Abcam, UK) were used to identify immunoreactivity. Visualisation of specific protein expression patterns was performed by chemiluminescence using either an EZ-ECL kit (Geneflow, UK) or Amersham ECL Plus™ Western Blotting Detection System (Amersham, UK) in conjunction with a Biorad Universal III Bioplex imager. ImageJ software™ was used for the densitometric analysis of the western blot bands. β-actin expression was used as a loading control in the western blot analysis.

### Assessment of Nitric Oxide or Hydrogen Peroxide Cytotoxicity

After 48 h in culture, SH-SY5Y cells in 96-well plates were exposed to experimental conditions. Medium was removed from all wells, cells were washed twice in DMEM and minimal medium or MSC conditioned medium containing the nitric oxide donor, DETA-NONOate (ranging from 0 to 800 μM; Fluorochem, UK) or hydrogen peroxide (ranging from 0 to 800 μM; Sigma-Aldrich, Gillingham, UK) was added for 6 h. Evaluation of cell survival was carried out using the MTT cell viability assay.

### MTT Cell Viability Assay

The supernatant was removed and replaced with Hank’s balanced salt solution (HBSS)/10% FCS containing 1 mg/ml 3-(4, 5-di methyl thiazol-2-yl)-2, 5-diphenyltetrazolium bromide (MTT; Sigma-Aldrich, Gillingham, UK). Cells were then incubated for 1 h at 37 °C, and subsequently, the HBSS/10% FCS/MTT solution was removed by inverting the plate and the plate left to dry. Dimethyl Sulfoxide (DMSO) (Sigma-Aldrich, Gillingham, UK) was then added to each well and the absorbance of the solution in each well read in a plate reader at 540 nm. Cell survival was assessed as a ratio of the amount of formazan production relative to the respective control. In all cases, control cultures grown throughout the experimental period in minimal medium were analysed and values for the experimental conditions divided by this value, in order to standardise results between experiments.

### Microscopy and Cell Quantification

Cells were visualised and images taken using a Motic AE2000 inverted phase contrast microscope, Moticam 10 camera and Motic images plus 2.0 software (Motic). At least 5 independent samples were included in the analyses. All cells were counted within five randomly assigned set areas distributed around the culture well.

### Statistical Analysis

The analysis was performed using GraphPad Prism (GraphPad Software Inc., USA). For all tests, values of *p* < 0.05 were considered statistically significant. At least three independent samples from each group were included in the analyses. Data between two groups were analysed using either unpaired *t* tests or Mann-Whitney *U* tests. Statistical comparisons for over two groups were analysed using either the Friedman test, one-way or two-way analysis of variance (ANOVA) with post hoc testing between groups where appropriate. Data are represented as mean ± SEM.

## Results

### Transfection of SH-SY5Y Cells with FXN siRNA Results in Decreased Frataxin Expression

**Fig. 1:**
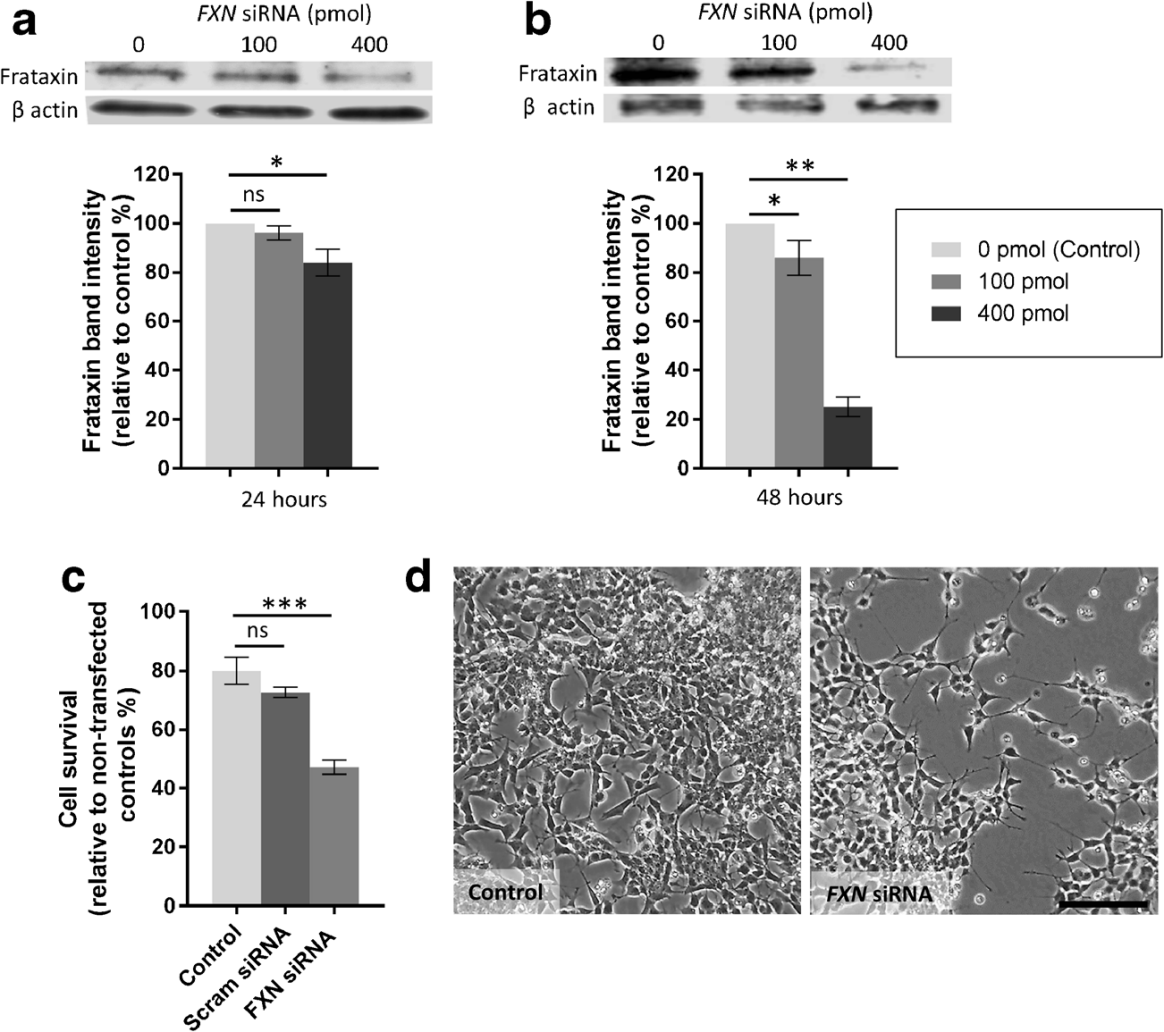
Frataxin expression in SH-SY5Y cells after *FXN* siRNA transfection. Western blotting images and corresponding densitometric analysis (integrated density) demonstrating expression of frataxin in SH-SY5Y cells following transfection using 100 or 400 pmol *FXN* siRNA after (**a**) 24 h in culture or (**b**) 48 h in culture. β-actin was used as a loading control. Results are expressed as mean percentage relative to H_2_O carrier transfected controls. **c** Percentage of SH-SY5Y cell survival following transfection with 400 pmol *FXN* siRNA, scrambled (Scram) siRNA or H_2_O carrier (control) for 48 h. **d** Representative images of SH-SY5Y cells 48 h post-transfection with 400 pmol *FXN* siRNA. ± SEM; *n* = 3; *p < 0.05; **p < 0.01; ***p < 0.001; *ns* not significant; *scale bar* = 100 μm

Preliminary experiments were performed transfecting SH-SY5Y cells using several concentrations of of *FXN* siRNA. Utilising western blotting and protein band densitometry, it was demonstrated that transfection using 100 or 400 pmol *FXN* siRNA (diluted in H_2_O) resulted in a reduction in the expression of the frataxin protein (Fig. 1a, b). 48 h post transfection with 100 pmol *FXN* siRNA resulted in an approximate 20% reduction in frataxin protein expression (*p* < 0.05; Fig. 1b). Transfection using 400 pmol *FXN* siRNA resulted in a 15% reduction in frataxin levels after 24 h of culture (*p* < 0.05; Fig. 1a) and 75% knockdown in frataxin protein levels following 48 h in culture (*p* < 0.01; Fig. 1b). β-actin was used as an internal western blot loading control. For all further experimentations, transfection was carried out using 400 pmol *FXN* siRNA.

### Transfection with FXN siRNA Decreases Survival of SH-SY5Y Cells

The number of viable cells in each group was calculated as a percentage normalised to a non-transfected control group. Forty-eight hours following transfection, five random fields per culture were counted and averaged. The transfection process itself caused cell death as can be seen in the reduction of live cells (approximate 20% reduction) in the H_2_O carrier or scrambled siRNA transfected groups (Fig. 1c). A significantly higher proportion of cell death was also seen amongst the *FXN* siRNA transfected cells (approximate 40% reduction) compared to either H_2_O carrier or scrambled siRNA transfected controls (*p* < 0.001; Fig. 1c, d).

### Sensitivity to Oxidative Stress

**Fig. 2:**
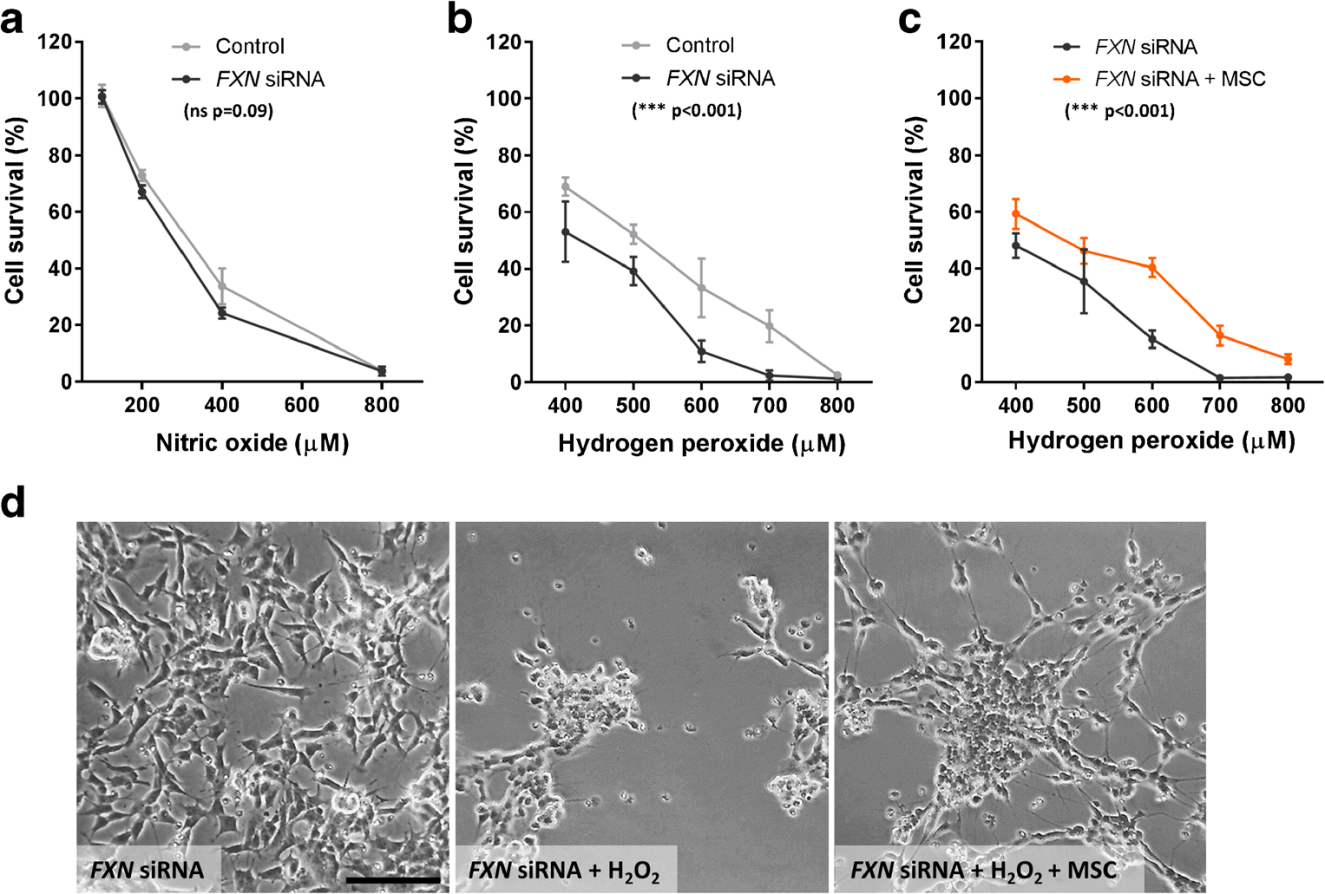
Susceptibility to nitric oxide and hydrogen peroxide mediated toxicity**.** Cell survival determined using an MTT viability assay in SH-SY5Y cells transfected with *FXN* siRNA or H_2_O carrier (control) following 6 h exposure to varying levels of (**a**) DETA-NONOate (Nitric oxide) and (**b**) hydrogen peroxide. **c** Cell survival of SH-SY5Y cells transfected with *FXN* siRNA exposed to minimal medium or MSC conditioned medium (MSC) and differing levels of hydrogen peroxide for 6 h. **d** Representative images of SH-SY5Y cells transfected with *FXN* siRNA exposed to minimal medium or MSC conditioned medium (MSC) with/without 6 h exposure to 600 μM hydrogen peroxide. Results are expressed as mean percentage value relative to control (0 μM hydrogen peroxide). ± SEM; *n* = 3; groups were compared using 2-way ANOVA. *** *p* < 0.001; *ns* not significant; *Scale bar* = 100 μm

Sensitivity of *FXN* siRNA transfected SH-SY5Y cells to oxidative stress was investigated using the 3-(4,5-dimethylthiazol-2-yl)-2,5-diphenyltetrazolium bromide (MTT) viability assay. Transfected cells were exposed to the nitric oxide donor DETA-NONOate in minimal medium for 6 h at concentrations ranging from 0 to 800 μM. Cell survival decreased with increasing concentrations of the nitric oxide donor. No significant difference was seen between the *FXN* siRNA transfected cells and the H_2_O carrier transfected control group (*p* = 0.09; Fig. 2a). Cells were also exposed to hydrogen peroxide in minimal medium for 6 h at concentrations ranging between 0 and 800 μM. Cell survival decreased with increasing hydrogen peroxide concentrations. There was also a significant difference between the *FXN* siRNA and H_2_O carrier transfected groups; the cells transfected with *FXN* siRNA were more sensitive to hydrogen peroxide over the concentrations tested (*p* < 0.001; Fig. 2b).

### Neuroprotection of Frataxin-Deficient Cells by MSC Conditioned Medium

The effects of MSC-secreted factors on the survival of *FXN* siRNA transfected SH-SY5Y cells was next determined. Cellular toxicitiy was mediated by exposing *FXN* siRNA transfected cells to hydrogen peroxide at concentrations ranging from 0 to 800 μM for 6 h in either minimal medium or minimal medium conditioned by MSCs (MSC conditioned medium). An MTT cell viability assay was performed. MSC conditioned medium significantly increased cell survival from hydrogen peroxide-mediated injury when compared to cells exposed to hydrogen peroxide in minimal medium alone (*p* < 0.001; Fig. 2c, d).

### Expression of Anti-oxidant Enzymes

**Fig. 3:**
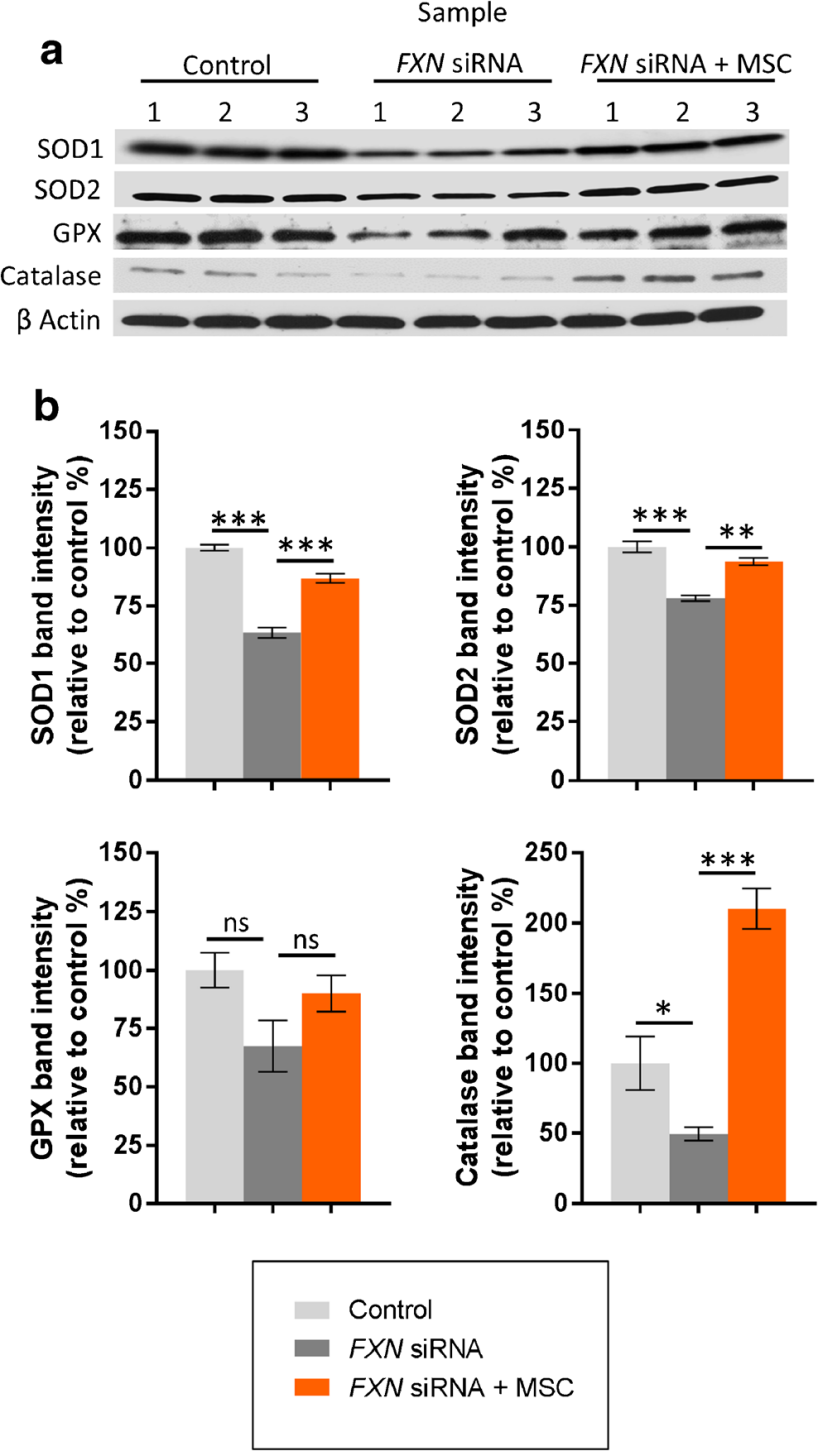
Expression of anti-oxidant enzymes. Western blotting images (**a**) and corresponding densitometric analysis (integrated density) (**b)** demonstrating protein expression of superoxide dismutase 1 (SOD1), superoxide dismutase 2 (SOD2), glutathione peroxidase 1 (GPX) and catalase in SH-SY5Y cells following transfection with H_2_O carrier (control) or *FXN* siRNA with/without exposure to MSC conditioned medium (MSC). Blots represent three separate cultures per condition. β-actin was used as a loading control. Results are expressed as mean percentage relative to H_2_O carrier transfected controls. ± SEM; *n* = 3; *p < 0.05; **p < 0.01, ***p < 0.001; *ns* not significant

Forty-eight hours post transfection, H_2_O carrier or *FXN* siRNA transfected SH-SY5Y cells were exposed to either minimal medium or MSC-conditioned medium for 24 h. Subsequently anti-oxidant enzyme expression was determined using western blotting and protein band densitometry. To ensure equal loading of cell lysates, β-actin was used as a loading control. Superoxide scavenging enzymes; SOD1 and SOD2 catalyse the dismutation of reactive superoxide anions into hydrogen peroxide and oxygen. Both SOD1 and SOD2 were expressed at significantly lower levels in the *FXN* siRNA transfected cells compared to H_2_O carrier transfected controls (*p* < 0.001; Fig. 3a, b). In addition, there was a significant decrease in the levels of the hydrogen peroxide scavenging enzyme catalase in *FXN* siRNA transfected cells compared to H_2_O carrier transfected controls (*p* < 0.05; Fig. 3a, b). Although lower, no significant change was observed for the hydrogen peroxide scavenging enzyme glutathione peroxidase 1 after transfection with *FXN* siRNA (*p* = 0.10; Fig. 3a, b).

Exposure of *FXN* siRNA transfected cells to MSC conditioned medium resulted in an amplification of both SOD1 and SOD2 expression (when compared to base minimal medium alone) to that approximately observed in H_2_O carrier transfected controls (*p* < 0.01; Fig. 3a, b). Exposure of *FXN* siRNA transfected cells to MSC conditioned medium also resulted in a non-significant elevation in glutathione peroxidase 1 expression and a significant amplification in the levels of catalase by approximately 4-fold when compared to cells cultured in minimal medium alone (being 2-fold greater than expressed in H_2_O carrier transfected controls) (*p* < 0.001; Fig. 3a, b).

### Frataxin, Regulators of Cell Survival, and Anti-oxidant Responses

**Fig. 4:**
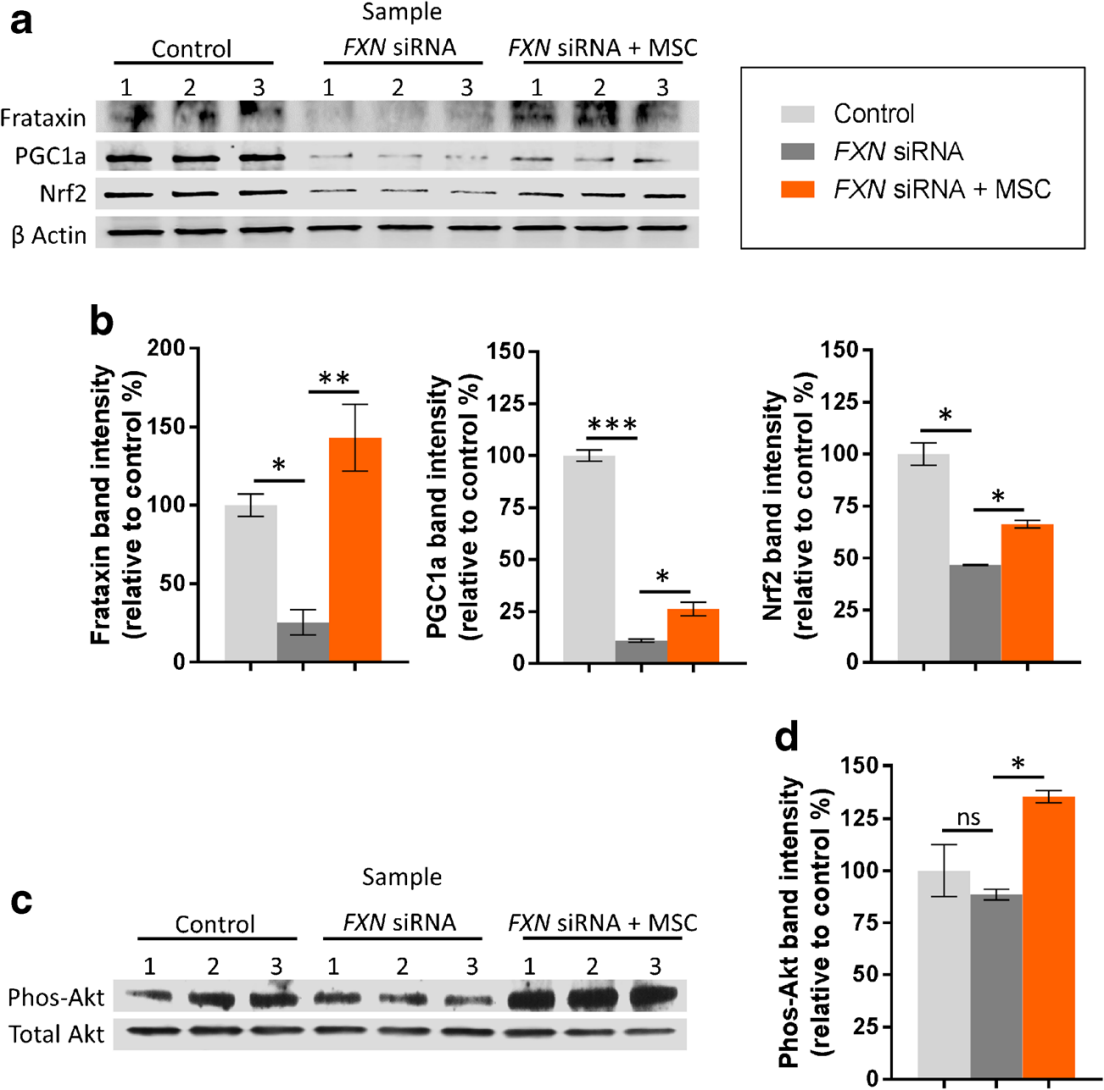
Expression of frataxin, regulators of cell survival, and anti-oxidant responses. Western blotting images (**a**, **c**) and corresponding densitometric analysis (integrated density) (**b**, **d**) (demonstrating protein expression of frataxin; peroxisome proliferator-activated receptor gamma coactivator 1-alpha (PGC1a); nuclear factor (erythroid-derived 2)-like 2 (Nrf2); and phosphorylated-Akt (phos-Akt) in SH-SY5Y cells following transfection with H_2_O carrier (control) or *FXN* siRNA with/without exposure to MSC conditioned medium (MSC). Blots represent three separate cultures per condition. β-actin or total Akt were used as loading controls. Results are expressed as mean percentage relative to H_2_O carrier transfected controls. ± SEM; *n* = 3; *p < 0.05; **p < 0.01, ***p < 0.001; *ns* not significant

Forty-eight hours post transfection, H_2_O carrier and *FXN* siRNA transfected SH-SY5Y cells were exposed to either minimal medium or MSC conditioned medium for 24 h. Subsequently, protein expression was detected using western blotting techniques. Image analysis using ImageJ software calculated that, in the presence of MSC conditioned medium, frataxin protein expression was significantly upregulated fivefold in *FXN* siRNA transfected cells when compared to cells cultured in minimal medium alone (*p* < 0.01) (Fig. 4a, b).

PGC1a and Nrf2 are key orchestrators of cellular anti-oxidant responses and their expression is reduced in frataxin-deficient cells [20, 21]. In line with other frataxin-deficient models, *FXN* siRNA transfected cells had significantly reduced protein levels of both PGC1a and Nrf2 when compared to respective H_2_O carrier transfected controls (*p* < 0.05; Fig. 4a, b). Conversely, exposure of *FXN* siRNA transfected cells to MSC conditioned medium resulted in a significant amplification in the levels of PGC1a and Nrf2 protein expression when compared to cells cultured in minimal medium alone (*p* < 0.05; Fig. 4a, b).

We also studied the influence of MSC conditioned medium on the PI3kinase/Akt pathway in *FXN* siRNA transfected SH-SY5Y cells. H_2_O carrier and *FXN* siRNA transfected cells were incubated with MSC conditioned medium or minimal medium for 6 h before lysis and analysis of phosphorylated-Akt levels (relative to total Akt) using western blotting and densitometric analysis. Phosphorylated-Akt levels did not differ between *FXN* siRNA transfected cells and H_2_O carrier transfected controls. However, MSC conditioned medium significantly increased the levels of phosphorlyted-Akt in *FXN* siRNA transfected cells relative to *FXN* siRNA transfected cells cultured in minimal base medium alone (*p* < 0.05; Fig. 4c, d).

### Cell Proliferation and Differentiation

**Fig. 5:**
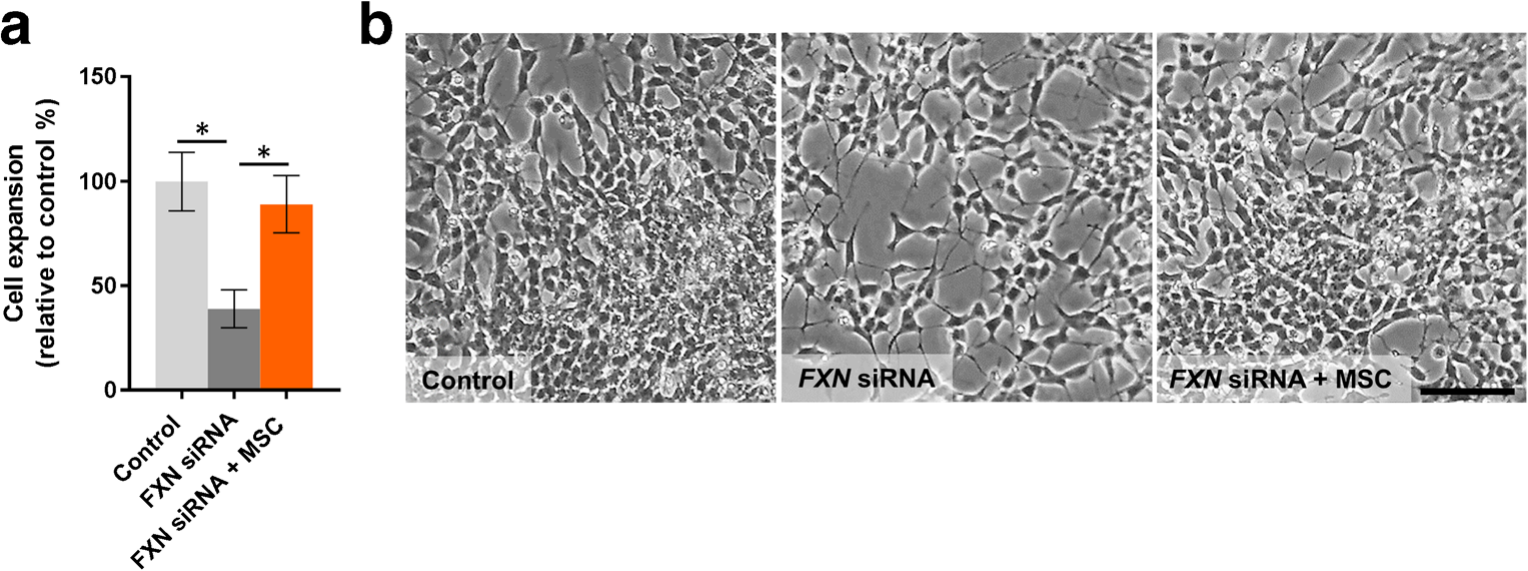
Cell expansion of *FXN* siRNA transfected SH-SY5Y cells. Cell expansion (**a**) and representative images of SH-SY5Y cells 8 days post transfection with H_2_O carrier control (control) or *FXN* siRNA with/without exposure to MSC conditioned medium (MSC) (**b**). Results are expressed as mean percentage relative to H_2_O carrier transfected controls. ± SEM; *n* = 5; *p < 0.05; *Scale bar* = 75 μm

**Fig. 6:**
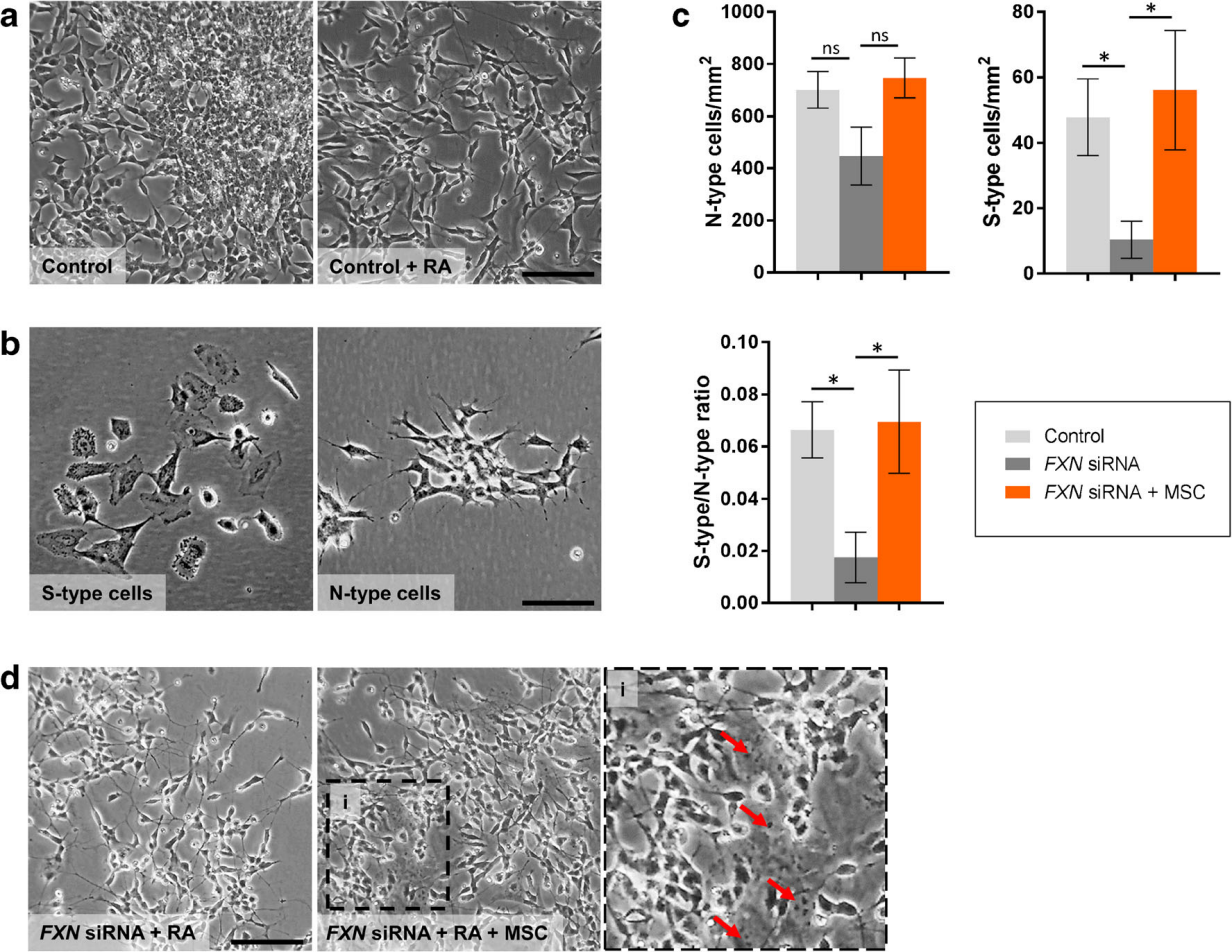
Differentiation of *FXN* siRNA transfected SH-SY5Y cells**. a** Representative images of SH-SY5Y H_2_O carrier transfected controls (Control) cultured for 8 days with or without addition of retinoic acid (RA). **b** Images depicting cells with either an N-type or S-type morphology. **c** The number N-type cells, S-type cells and the S-type/N-type ratio following transfection with H_2_O carrier (control) or *FXN* siRNA and differentiation with RA with/without exposure to MSC conditioned medium (MSC). **d** Representative images of RA differentiated SH-SY5Y cells following transfection with *FXN* siRNA with/without exposure to MSC conditioned medium (MSC). *Red arrows* indicate the position of S-type cells. ± SEM; *n* = 5; *p < 0.05; *ns* not significant; *Scale bars* = 100 μm (**a**, **d**), 50 μm (**b**)

*FXN* siRNA transfected cells displayed a significantly slower rate (approximately 50% decrease) in expansion over 8 days in culture when compared to H_2_O carrier transfected controls (*p* < 0.05; Fig. 5a, b). Under differentiation culture conditions using exposure to retinoic acid, the large majority of H_2_O carrier transfected control cells over time became relatively polar, with branching of long neurites, characteristic of the N-type (Neuronal) phenotype (Fig. 6a, b). A significantly smaller number (approx. 6%) of flat rounder cells were also observed, typical of S-type (Schwann) cells (Fig. 6b). The frequency of N-type cells after 8 days of culture, although lower, did not significantly differ between *FXN* siRNA and H_2_O carrier transfected cells (*p* = 0.17; Fig. 6c), however, there was a significant decrease in both the levels of S-type cells and subsequently the S-type/N-type ratio in *FXN* siRNA transfected cells compared to H_2_O carrier transfected controls (*p* < 0.05; Fig. 6c).

Investigating the effects of MSC conditioned medium on both cell expansion and differentiation of *FXN* siRNA transfected cells, we found addition of MSC conditioned medium significantly increased the rate of cellular expansion when compared to cells exposed to minimal medium alone (*p* < 0.05; Fig. 5a, b). Equally, exposure to MSC conditioned medium under differentiation conditions, the levels of both S-type cells and the S-type/N-type ratio were significantly increased and restored to those seen in the H_2_O carrier transfected control group (*p* < 0.05; Fig. 6c, d). We found no significant changes in either expansion or numbers of N- and S-type cells in the H_2_O carrier transfected control group exposed to MSC conditioned medium (data not shown).

## Discussion

Oxidative damage and inhibition of mitochondrial function may be key determinants of cellular damage in FA. Cells deficient in frataxin have a greater sensitivity to oxidative stress and also have an impaired ability to recruit anti-oxidant defences [22, 23]. At present, there are no licenced therapies for disease modification in FA. However, several strategies have been developed aiming to reduce the impact of frataxin deficiency, including trialling agents that protect against oxidative damage and mitochondrial respiratory chain defects (e.g. idebenone, pioglitazone and deferiprone) [24, 25]. Strategies to increase frataxin expression using drugs such as recombinant erythropoietin, interferon-gamma, nicotinamide, insulin-like growth factor-1 (IGF-1), resveratrol and histone deacetylase enzyme inhibitors (HDACi) are also being tested [26–31].

The human SH-SY5Y cell line originates from a neuroblastoma patient and these cells are widely used in neurological studies. SH-SY5Y cells are multipotent precursor cells that give rise to distinct neural crest cell lineages. The SH-SY5Y cell line has particular relevance to FA, since many cells affected in the disease derive from the neural crest, and the pathophysiology of FA may be caused by defects to shared precursor cells [17]. Three different cellular phenotypes of SH-SY5Y cells have been identified: neuronal (N type); Schwann (S type); and intermediary (I type). More specifically, SH-SY5Y cells have the ability to differentiate into neuronal and Schwann cell progenitors, with prolonged exposure to retinoic acid [32]. We have developed a cellular model of frataxin deficiency in the SH-SY5Y cell line using frataxin knockdown by siRNA technology. Since symptomatic patients have less than 35% of frataxin levels compared to that found in healthy controls [33], the aim was to develop a model to accurately represent this. Forty-eight hours post exposure to 400 pmol of *FXN* specific siRNA down-regulated frataxin protein levels to approximately 25% of that found in H_2_O carrier transfected controls. Importantly, SH-SY5Y cells with reduced frataxin expression showed a similar phenotype to other FA models, namely: reduced cellular survival and increased susceptibility to oxidative stress.

In line with previous reports in cellular and animal models of FA, frataxin deficiency was associated with reduced protein levels of PGC1a and Nrf2; both molecules being key orchestrators of cellular anti-oxidant responses [34, 35]. Frataxin knockdown also reduced expression of SOD1 and SOD2 in SH-SY5Y cells. Cells derived from patients with FA have previously demonstrated SOD deficiencies [7]. SOD1 and SOD2 are both involved in dismutation of reactive superoxide anions to prevent the build-up of the highly toxic metabolite peroxynitrite. Interestingly, in this study, unlike previous reports in FA patient cells [7], we did not find an increased sensitivity to nitric oxide-mediated toxicity post frataxin knockdown. A lack of increased sensitivity to nitric oxide may be a possible consequence of alternate compensatory anti-oxidant mechanisms.

*FXN* siRNA transfected cells exposed to hydrogen peroxide showed a significant reduction in survival compared to transfected controls. Increased sensitivity to hydrogen peroxide in frataxin-deficient cells has been previously noted in FA models [6]. A possible explanation for this increased sensitivity to hydrogen peroxide may be caused by the apparent reductions in catalase levels. Reductions in hydrogen peroxide scavenging enzymes have been reported in cells derived from patients with FA and are thought to contribute to increased susceptibility to oxidative stress in these models [9]. Indeed, pathological studies of FA have demonstrated that oxidative damage and increased sensitivity to oxidative stress may be important contributors to the pathogenesis of the disease. In FA, low frataxin levels and defective anti-oxidant defences causes iron overload through depletion of iron-sulphur cluster synthesis [36]. Reactive free iron promotes Fenton chemistry, producing superoxide and hydrogen peroxide, which in turn destroys more iron-sulphur clusters [36]. High levels of lipid peroxidation products, such as malondialdehyde, which are formed by the action of oxygen radicals (including hydroxyl (OH) and peroxynitrite (ONOO)) on lipid membranes are also observed in FA [37]. These reactive aldehydes are themselves cytotoxic, having the capacity to both inhibit DNA, RNA and protein synthesis and disrupt membrane structures [38]. They can also perpetuate oxidative stress by elevating mitochondrial reactive oxygen species and inhibiting anti-oxidant enzymes [39], which may, in part, explain reductions in anti-oxidant molecules in frataxin-deficient SH-SY5Y cells. Strategies to reduce oxidative injury may therefore have important therapeutic implications for the disease.

Bone marrow MSC transplantation regimens have been studied as potential therapies for a number of neurological disorders. Human MSCs have been shown to have a range of neuroprotective and neuro-regenerative properties. Studies have shown that MSCs release a plethora of potentially neuroprotective growth factors including brain-derived neurotrophic factor (BDNF), IGF-1, neurotrophin-3 [13, 15], stem cell factor (SCF) and granulocyte-colony stimulating factor (G-CSF) [40], some of which have already shown promising therapeutic potential for FA [31, 41, 42]. Regarding disorders characterised by elevations in oxidative stress, MSCs can secrete anti-oxidant molecules, which directly promote cellular survival following oxidative stress by reducing toxic reactive oxygen species; and may also induce nervous system cells to increase endogenous production of anti-oxidant defences [18, 43].

In the current study, bone marrow-derived MSCs were studied in order to determine if they secrete factors that may offer protection against the cellular dysregulation induced by frataxin deficiency. Importantly, in agreement with previous reports [7, 9, 12], we showed that exposure of *FXN-*deficient cells to MSC conditioned medium increased frataxin protein expression. This is potentially due to the presence of several known inducers of frataxin expression, including IGF-1, SCF and G-CSF within MSC conditioned medium [42, 44]. Clinical therapies that are able to elevate frataxin levels, in theory, may be able to re-establish normal cellular function by increasing frataxin levels to a threshold at or above that found in asymptomatic heterozygous carriers of the GAA repeat expansion [33]. Several agents have been tested for this purpose, but there has been mixed success in their ability to increase frataxin levels when tested in patients with FA [28–30, 45]. In line with increases in frataxin, exposure to MSC conditioned medium also elevated the expression of molecules associated with frataxin’s anti-oxidant functions including PGC1a, Nrf2, superoxide dismuting enzymes (SOD 1 and 2) and the hydrogen peroxide-scavenging enzyme catalase. Since MSC conditioned medium protected SH-SY5Y cells from hydrogen peroxide toxicity, the marked elevation in catalase expression may be a major contributor to neuroprotection in the model. The increase in catalase induced by MSC conditioned medium is of relevance, since hydrogen peroxide scavenging has been shown to rescue frataxin deficiency in a FA *Drosophila* model [6].

In an attempt to further identify molecular mechanisms underlying the protective role of MSCs, we investigated PI_3_kinase pathway signalling in *FXN* siRNA transfected cells. We have previously shown that intact PI_3_kinase/Akt pathway signalling is particularly important in the survival of human FA-fibroblast cells against oxidative stress [7]. Moreover, IGF-1, a potential therapeutic currently being tested in FA [31], has been shown to normalise frataxin levels in frataxin-deficient neurons and astrocytes through the Akt/mTOR signalling pathway [44]. Interestingly, SH-SY5Y cells deficient in frataxin displayed no block in Akt signalling; however, in the presence of MSC conditioned medium, a large increase in Akt signalling was observed. MSC-mediated Akt activation in frataxin-deficient cells may therefore play a critical role in maintaining homeostasis and survival against cellular stress.

When investigating the effects of frataxin knockdown on cellular proliferation and differentiation, we found a marked reduction in both cell expansion and S-type (Schwann) cell formation. Previous in vitro studies using human Schwann cell lines show frataxin knockdown blocks cell cycle progression at G_2_M; this is followed by an upregulation of inflammatory/apoptotic genes and cell loss [46]. Indeed, with a deficient anti-oxidant system, the build-up of peroxynitrite and subsequent increase in lipid peroxidation products can lead to inhibition of DNA synthesis [38], thus a block in cell proliferation. Pathologically, lack of myelination of large sensory axons and loss of dorsal root ganglia cells is found in patients with FA [47]. Myelinating Schwann cells help maintain axon integrity, thus several studies have suggested that Schwann cells could be defective in FA and that degeneration of peripheral neurons occurs as a secondary consequence [46, 48]. Post-mortem analysis of sural nerves of patients who had FA shows a lack of myelinated fibres (hypomyelination) whereas axons are present in normal numbers [48]. Histological investigations on the dorsal roots in post-mortem samples also show differences in the myelination of thin fibres and fewer numbers of Schwann cells [48]. This provides evidence towards possible defective Schwann cells or Schwann cell precursors in FA [17], as was also apparent in the frataxin-deficient SH-SY5Y cell model. Exposure to MSC conditioned medium significantly reversed the inability of frataxin-deficient SH-SY5Y cells to differentiate down the S-type cell lineage. MSCs are known to produce several factors recognised for aiding differentiation of SH-SY5Y cells, including both BDNF and IGF-1 [49, 50]. MSCs can also promote cell survival and proliferation of Schwann cells in vitro, and in vivo stimulate peripheral nerve repair through increased the generation of Schwann cells [51]. Together, our data suggest that MSCs may have the therapeutic capacity to help stimulate the maturation and differentiation of Schwann cells to aid myelin/axonal repair in FA.

## Conclusion

In conclusion, we have used frataxin knockdown in SH-SY5Y cells as a cellular model for FA. *FXN* siRNA transfected SH-SY5Y cells have proved to be a useful model for FA since many of the biochemical properties of true FA patient-derived cells are replicated. Knockdown of frataxin expression to 25% of that seen in transfected controls was achieved and the phenotype of cells revealed (i) decreased cellular viability, (ii) increased susceptibility to hydrogen peroxide-induced oxidative stress, (iii) reduction in expression of key anti-oxidant molecules and (iv) a deficiency in both cell proliferation and differentiation. The FA cellular model was used to study potential neuroprotective effects of MSCs. We show MSC-secreted factors were able to protect against the cellular dysregulation induced by frataxin deficiency. The demonstration that factors produced by MSCs can regulate frataxin expression and restore cellular homeostasis suggests that they may have potential therapeutic benefits for patients with FA.
